# Stress Assignment Errors in Surface Dyslexia: Evidence from Two Italian Patients with a Selective Deficit of the Orthographic Input Lexicon

**DOI:** 10.1155/2015/769013

**Published:** 2015-04-30

**Authors:** Alessia Folegatti, Lorenzo Pia, Anna Berti, Roberto Cubelli

**Affiliations:** ^1^SpAtial, Motor & Bodily Awareness (SAMBA) Research Group, Psychology Department, University of Turin, Via Po 14, 10123 Turin, Italy; ^2^Neuroscience Institute of Turin (NIT), University of Turin, Via Po 14, 10123 Turin, Italy; ^3^Department of Psychology and Cognitive Sciences, Trento University, Italy

## Abstract

Surface dyslexia designates a selective impairment in reading irregular words, with spared ability to read regular and novel words, following a cerebral damage usually located in the left dominant hemisphere. In Italian language, which is regular at the segmental level, surface dyslexia is characterized by stress assignment errors. Here we report on two cases of Italian surface dyslexic patients who produced stress assignment errors, mainly in reading irregular words. In reading nonwords they usually applied the regular stress pattern. Both patients were also impaired in lexical decision and in semantic discrimination tasks when the processing of homophones was required. Our patients' performance relied almost exclusively on the phonological coding of the stimulus, revealing a deficit in accessing the orthographical input lexicon. In addition, one patient showed a cerebral lesion limited to the right thalamus, providing evidence of a possible role of the right hemisphere in the reading process.

## 1. Introduction

Surface dyslexia is an acquired reading disorder characterized by a selective impairment in reading irregular familiar words, coupled with a preserved ability to read regular words, unknown words, and nonwords [1]. Typically, it results from a cerebral lesion located in the left, dominant hemisphere [2].

According to the dual-route model [3], surface dyslexia is assumed to reflect the predominant use of the grapheme-to-phoneme conversion procedure due to a deficit of the lexical route, that is, a deficit in accessing the stored lexical knowledge. Different subtypes depending on the loci of impairment through the lexical route have been reported in both acquired [4] and developmental [5] surface dyslexia. All the subtypes of surface dyslexia show the typical pattern of regularization errors in reading aloud. However, they are associated with different performance in tasks requiring lexical decision with pseudohomophones and homophone comprehension [6].

Surface dyslexia has been often described in languages with opaque orthography, for example, English [7, 8] or French [9]. In such languages, the phonological forms corresponding to the orthographic segments can be ambiguous; for example, in English the letter sequence <EA> is pronounced differently in the words* head*,* beat*,* heart*, and* great*; similarly, in French the letter sequence <EN> is read differently in the words* chrétienté* [Christianity] and* impatiente* [impatient]. For a number of words, the correct pronunciation cannot be derived using the grapheme-to-phoneme (GTP) conversion rules; therefore, to be read flawlessly, “exception” or “irregular” words have to be learnt by heart and stored in the lexicon with their particular orthography to phonology mappings. Typically, surface dyslexic patients produce regularization errors, with irregular words pronounced with the statistically typical GPT correspondences, for instance,* listen* →* liston* [3] or* have* →* hayve* [8]. This kind of errors, which can be considered “legitimate alternative readings of components” responses [1], has been reported also in nonalphabetic languages, for example, Chinese [10] and Japanese [1].

In languages with transparent orthography, like Spanish [11], surface dyslexia is mainly characterized by homophone confusions and errors in lexical decision with pseudohomophones. Overall, the pattern of impairment depends on the features of the language; for instance, in Hebrew most errors are incorrect vowel pronunciations in reading unvoweled homographs [12], whereas in Italian most errors are stress assignment errors [13, 14].

In Italian, the GTP mapping is regular. Almost all phonemes are represented by a grapheme constituted by a single letter (e.g., <B> → /b/; <F> → /f/). In a small set of cases, the selection of the correct phoneme depends on the following letter (e.g., the letter C is read as /k/ when followed by consonants or by the vowels A, O, and U or as /c/ when followed by the vowels E and I). At the segmental level, therefore, Italian is a very transparent language, with all phonemes being derived from letters by means of general conversion rules. Nevertheless, Italian written language is opaque at the suprasegmental level. Stress position is obligatorily marked only when stress falls on the last syllable (e.g.,* umiltà* [humbleness]). In the other cases, stress position is not marked diacritically; even it is completely unpredictable [15]. Therefore, reading words having more than two syllables requires access to stored lexical information. Consider, for instance, the word pairs* colline* (hills) and* polline* (pollen),* legato* (bound) and* fegato* (liver): they differ only in the first letter but have stress falling on different syllables, the penultimate and the antepenultimate, respectively. About 80% of three-syllabic words are stressed on the penultimate syllable, while about 18% are stressed on the initial (antepenultimate) syllable. Given the statistical distribution, stress on the penultimate syllable is usually referred to as the “regular” pattern [16].

Italian patients with surface dyslexia have been often reported. Whereas in some cases stress errors appear to be due to a deficit not limited to reading [17] or to a more general cognitive disorder [18, 19], in two dyslexic patients showing a selective deficit of stress assignment it is possible to locate the functional impairment on the lexical route. Patient CLB described by Miceli and Caramazza [13] showed jargon aphasia and was impaired in all verbal tasks. In reading aloud he was correct at the segmental level but made errors involving stress assignment (*'macchina* [car] →* ma'cchina* [nonword]). Since he produced very few errors in lexical decision and in comprehension tasks, his stress assignment errors appear to be reflecting a deficit at the level of the phonological output lexicon. Patient CA [14] was affected by slowly progressive aphasia with frequent anomia and circumlocutions in spontaneous speech. In both naming and comprehension tasks he produced semantic errors, whereas in reading word aloud he made a number of stress assignment errors (*'nevica* [it snows] →* ne'vica* [nonword]). His overall pattern of performance is consistent with a deficit affecting both the semantic system and the phonological output lexicon.

To our knowledge there are no Italian dyslexic patients described in literature showing deficits due to a selective impairment at the orthographic input lexicon. In the present paper, for the first time, we report on two Italian patients with surface dyslexia who showed almost exclusively errors in assigning the correct stress pattern and in processing nonhomographic homophones stimuli, which is the pattern of performance consistent with an impaired orthographic input lexicon. Moreover, noticeably, while one patient presented with a cerebral lesion involving the left, dominant hemisphere, the other one had a lesion limited to the right thalamus. This instance testifies an involvement of the right hemisphere in the reading process and suggests that lesions affecting different cerebral areas may underlie similar patterns of impairment.

## 2. Methods

### 2.1. Participants

Two brain-damaged patients with evidence of surface dyslexia participated in the study.

**Figure 1:**
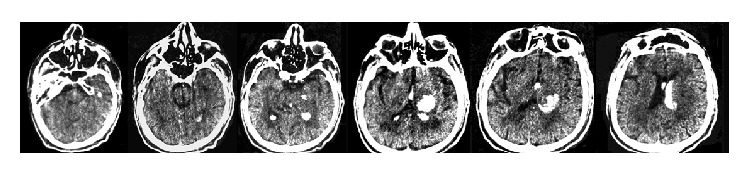


The first patient, BL, is a right-handed, 69-year-old man, with 13 years of formal education, who suffered a hemorrhagic damage to the right hemisphere. A CT scan revealed a right thalamic intraparenchymal hemorrhage with an expansion to the third and the lateral ventricles (see Figure 1).

At the admission, the first neurological examination revealed confusional state, dysarthria, and left brachiofacial hemiplegia. At the time of testing (53 days after the onset of the lesion), no signs of neglect were detected on screening tasks: line cancellation (0/35 omissions) [20], bell cancellation (2/35 omissions) [21], and picture copying [22]. Verbal production was normal as well as auditory comprehension (Token Test: 34/36). However, rather surprisingly, when required to read words and nonwords, BL produced several stress assignment errors that were absent in spontaneous speech (e.g., ta'lora [sometimes] → ‘talora [nonword]). No other cognitive disorders were present.

The second patient, MA, is a 76-year-old male, with 5 years of formal education (a normal educational level for Italians of his age), who was referred to the hospital following the sudden appearance of expressive disorders. A CT scan showed an ischemic lesion involving the frontal parasagittal area and the temporooccipital area of the left hemisphere. When tested, two months after the onset of the lesion, he showed normal auditory comprehension but defective verbal production. On a standardized battery for aphasic disorders (BADA) [23], he was impaired on picture naming (correct responses 19/30, 63%) and direct repetition (34/45, 76%). Errors revealed impaired lexical access (anomia and circumlocutions) and difficulties in producing the correct phonological sequence of individual words (phonemic paraphasias and neologisms). In most cases, the frequent attempts of self-correction were successful. The processing of visual orthographic information appeared to be less impaired. Lexical decision of written stimuli was mildly defective (68/80, 85%), while reading words (85/92, 93%) and nonwords (42/45, 93%) aloud was quite normal. However, at variance with spontaneous speech where errors involved phonological segments, most reading errors involved only stress assignment (e.g., os'sia [that is] → 'ossia [nonword]; 'mandorla [almond] → man'dorla [nonword]). Nonverbal abilities were normal.

Both patients gave their verbal informed consent to participate in the study, which was approved by the Bioethics Committee of the University of Turin and was performed in accordance with the ethical standards laid down in the Declaration of Helsinki 2013.

### 2.2. Materials

To investigate reading abilities and orthographic processing, the two patients underwent the battery proposed by Sartori [24, 25]. To evaluate lexical decision and reading words and nonwords aloud, a list of 40 words and 40 pronounceable nonwords was used (subtest 4 of Sartori's battery). The words were controlled for length (4 or 7 letters), frequency, and concreteness; the nonwords were derived from the word stimuli by substituting one letter. To evaluate further the ability to read words, a second list was employed (subtest 7 of Sartori's battery). It comprises 80 words that were controlled for length (from 4 to 7 letters) and grammatical class (nouns, adjectives, verbs, and functors).

The battery comprises also subtests for letter recognition, semantic categorization, and comprehension of nonhomographic homophonic stimuli. Letter recognition was evaluated by asking the patients to name a list of 21 letters and, in a second task, to judge whether two letters, presented one in capital and one in lower case, were the same or not (e.g., rR or Gd). Semantic processing was tested by means of categorization tasks; patients were asked to classify written nouns as denoting animals or not (easy semantic decision) or as four-legged or not four-legged animals (hard semantic decision). Finally, two different tasks were used to assess homophone comprehension. In Italian, nonhomographic homophonic word pairs are very few (e.g., hanno [they have], anno [year]; cieco [blind], ceco [Czech]). Therefore, the contrasting stimuli were obtained, in the first task, by inserting an apostrophe within a word to generate a noun phrase (e.g.,* l'una* [the one] from* luna* [moon]) and, in the second task, by splitting the noun into two unrelated words (e.g.,* di vino* [of wine] from* divino* [divine]). In the first subtest (word comprehension), the patients had to choose out of four alternatives the correct meaning of nonhomographic homophones (e.g.,* l'ago* [the needle],* lago* [lake]). One of the three incorrect choices would be appropriate if the target word was confused with its homophone. In a second subtest (sentence discrimination), the patients were given short homophonic sentences, such as* I monaci abitano nel convento* [the monks live in a monastery] or* I monaci abitano nel con vento* [the monks live in with wind], and asked to indicate whether the sentence was written correctly or not.

To assess the ability to read words with different stress position, three different lists were used (lists are reported at the end of the paper). In all lists, half the stimuli had stress on the penultimate syllable (the most frequent stress position in Italian language) and half on the antepenultimate syllable. The first list was included in the Sartori battery [24] and comprised 60 three-syllable words matched for frequency and grammatical class (subtest 8 of Sartori's battery). The second list was derived from Miceli and Caramazza [13] and comprised 120 words, controlled for length (three and four syllables), grammatical class (nouns, adjectives, and verbs), and frequency (high and low). To investigate further the access to orthographic knowledge a third list was used; it comprised 10 noun pairs which share the same number of letters and the same two ending vowels but have different stress position [14]. For example, the nouns* sedia* [chair] and* bugia* [lie] end with the same sequence of letters <ia> but have different stress pattern. In the former noun, which represents the most frequent pattern in Italian, stress is on the syllable preceding the two final letters that constitute a diphthong (/ja/); in the latter noun, stress is on the vowel “i,” with the two ending letters representing a hiatus and pronounced as two distinct syllabic nuclei (/ia/). These lists are provided in the appendix.

Finally, a list of nonwords was given to test the effect of stress neighborhood [26]. In Italian language stress position reveals formal regularities related to the noun ending letters. For example, most words ending with -*olo* have antepenultimate stress (e.g., '*tavolo* [table]), whereas most words ending with -*ume* have penultimate stress (e.g.,* vo'lume* [volume]). The ending letters appear to influence also stress assignment in nonword reading: consistent with the stress neighborhood (words with the same final sequence and the same stress pattern), meaningless stimuli like* rocolo* and* aldume* tend to be read as having stress on antepenultimate and penultimate syllable, respectively (e.g., [27, 28]). A list of 50 nonwords was derived from 50 words with many “stress neighbors” by substituting one of the first letters. The stimuli were classified as expected to be read with stress position either on the antepenultimate or on the penultimate syllable, depending on the stress neighborhood of the original words. The list of nonwords was presented twice to MA.

## 3. Results

As shown in Table 1, both patients were able to process orthographic stimuli, to recognize single letters, and to categorize written words. Their performance on these tasks was flawless or just below normal limits [25]. However, both patients appear unable to process homophonic nonhomographic stimuli, that is, when the use of the phonology to orthography conversion rules does not allow accomplishing tasks requiring the access to lexical knowledge. In word comprehension and sentence discrimination, the performance of both patients was at chance level.

As reported in Table 2, both patients produced very few visual errors in reading words and nonwords. No lexical effect was present; nonwords were read as accurately as familiar words. Further, no effects of lexical variables such as concreteness, frequency, and grammatical class were found in reading words. Overall, this pattern of performance is consistent with a preserved ability in converting orthography to phonology when reading regular stimuli. However when the correct phonology cannot be derived from applying the GTP conversion rules, as in reading the lists of polysyllabic words and nonwords described above, both patients showed a clear effect of stress position (Table 3). Indeed, they read words with penultimate stress more accurately than words with antepenultimate stress (BL: *χ* ^2^ = 9.25; *P* < 0.005; MA: *χ* ^2^ = 16.15; *P* < 0.001). Most errors were stress displacement (Table 4), with the less frequent antepenultimate stress words read with stress on the penultimate syllable. Also in reading nonwords, patients assigned stress following the distributional information, according to which the penultimate stress is dominant in Italian. Almost all stimuli were read with stress on the penultimate position, either nonwords with a stress neighborhood mainly composed of antepenultimate stress words or those with a majority of penultimate stress neighbors.

The tendency to read words by assigning the most frequent stress pattern is confirmed by the performance in reading words ending with “i” plus vowel (e.g., -ia). BL read correctly 7/10 (70%) words with diphthong and 5/10 (50%) words with hiatus; MA read 8/10 (80%) words with diphthong and 4/10 (40%) words with hiatus. Again, most errors involved only stress position (e.g.,* funi'via* [cableway] →* fu'nivia*).

## 4. Discussion and Conclusion

In the present paper, we reported on two Italian brain-damaged patients with surface dyslexia. Their pattern of performance was characterized by regularization errors at the suprasegmental level and difficulties in tasks requiring the comprehension of homophones and the discrimination between words and pseudohomophones.

Both patients appeared to rely exclusively on the phonological coding of the stimuli. Within the framework of the dual-route model [3], their pattern of performance is consistent with a deficit involving the lexical route and the privileged use of the sublexical procedure. The greater accuracy in reading words with penultimate stress than in reading words with antepenultimate stress and the tendency to read nonwords by assigning the most common stress pattern suggest that the sublexical procedure can assign stress using the relative distribution of different stress patterns in the language [16]. Further, since the two patients showed errors with homophones and pseudohomophones, their reading performance can be accounted for by assuming a deficit at level of the orthographical input lexicon [6].

The individual neuropsychological picture confirms the functional locus of their impairment. BL showed neither aphasic disorders nor visual-perceptual impairments. Semantic knowledge and phonological lexicon appeared to be preserved. It follows that his deficit should be located at the level of orthographic input lexicon only. MA, on the contrary, was aphasic: the types of errors he produced in naming and repetition tasks revealed a disorder at the level of the phonological lexicon. To verify whether also orthographic lexical lexicon was impaired or not, MA underwent a spelling decision test [29] where he was asked to discriminate between a word and a pseudohomophone distracter (e.g., igiene [hygiene] and igene [nonword]; cuoco [cook] and quoco [nonword]). MA produced 16/44 (36.4%) errors, thus confirming an impaired orthographic knowledge.

It is the first time that Italian patients with surface dyslexia due to a disorder at the level of the orthographic input lexicon are reported. The present findings are striking also because one of our patients, BL, showed a cerebral lesion limited to the right thalamus and sparing the other brain structures (see Figure 1). We have no reason to assume that the patient's linguistic skills were located in the right hemisphere; he was right-handed and the right lesion affected neither his oral expression and comprehension nor his writing ability. The pattern of performance shown by this patient suggests a possible role of the right hemisphere in the reading process, which appears to be functionally similar to that of the left hemisphere. This finding is consistent with neuroimaging evidence for a bilateral activation in the head of the caudate nucleus and the anterior thalamus in reading low frequency words [30]. Independently of the side of the cerebral lesion therefore, damage to these areas can, at least in part, impair reading when lexical access is needed. The pattern of performance shown by BL fits perfectly this prediction. One can assume that damage to the right hemisphere prevents orthographic information stored in the “visual word form area” of the left hemisphere, particularly in the occipitotemporal sulcus [31], from being accessible anymore.

## Figures and Tables

**Table 1:** Performances of patients BL and MA on Sartori's battery [20]: correct responses.

Tasks	Patient BL	Patient MA
Letter naming	21/21 (100%)	19/21 (91%)
Letter discrimination	49/54 (91%)	52/54 (96%)
Lexical decision	64/80 (80%)	68/80 (85%)
Semantic decision		
Easy	38/40 (95%)	40/40 (100%)
Hard	36/40 (90%)	38/40 (95%)
Homophone processing		
Word comprehension	19/32 (59%)^∗^	18/32 (56%)^∗^
Sentence discrimination	17/32 (53%)^∗^	16/32 (50%)^∗^

^∗^Chance level.

**Table 2:** Patients' performance in reading words and nonwords (correct responses).

Tasks	Patient BL	Patient MA^∗^
Words	117/120 (97%)	198/212 (93%)
Nonwords	35/40 (87%)	79/85 (93%)

Stimuli included 120 words and 40 nonwords taken from subtests 4 and 7 of Sartori's battery [20]. ^∗^MA was given also 92 words and 45 nonwords taken from BADA [19].

**Table 3:** Correct responses (%) in reading aloud stimuli with different stress position (Lists 1 and 2 reported in the appendix).

Stimuli	Patient BL^∗∗^	Patient MA
Words		
Penultimate syllable	83/90 (92%)	82/90 (91%)
Antepenultimate syllable	68/90 (76%)	60/90 (67%)
Nonwords		
Penultimate syllable^∗^	16/25 (64%)	35/40 (78%)
Antepenultimate syllable^∗^	2/15 (13%)	4/60 (7%)

^∗^Responses consistent with the predicted stress position (for normal readers see [27, 28]). ^∗∗^For incidental reasons, BL was given only 40 nonwords.

**Table 4:** Type of errors produced by patients BL and MA in reading words and nonwords.

Tasks	Patient BL		Patient MA	
	Words	Nonwords	Words	Nonwords
Stress errors	21 (72%)	15 (68%)	31 (82%)	48 (79%)
Segmental errors	6 (21%)	6 (27%)	5 (13%)	4 (6%)
Mixed errors	2 (7%)	1 (5%)	2 (5%)	9 (15%)

## Appendix

Lists of words with different stress patterns used to assess the ability to read words with different position are presented as follows.

*List 1*

Profilo
Felice
Replicare
Ruvido
Famiglia
Prendere
Cucciolo
Costoso
Italiano
Includere
Passivo
Macchina
Addetto
Sillaba
Facile
Fiutare
Giovane
Chiudere
Milione
Funebre
Chirurgo
Vicino
Friggere
Piantare
Minimo
Pollaio
Cellula
Esempio
Soffocare
Lacero
Sbagliare
Lettera
Giungere
Libero
Appetito
Invadere
Materno
Lasciare
Candido
Sporgere
Dottore
Battere
Possibile
Sperare
Monello
Predire
Ripido
Rompere
Maggiore
Fortuna
Passero
Nervoso
Cucinare
Ordine
Diventare
Camera
Tingere
Orfano
Lucente
Genere.

*List 2*

Nuvole
Patate
Medicina
Camera
Cortile
Genitori
Simboli
Genere
Polvere
Numero
Parole
Tesoro
Denaro
Secoli
Favore
Cinema
Lettera
Mattina
Colore
Pagina
Tavolo
Regina
Natura
Pericolo
Cintura
Carcere
Pecore
Costume
Portici
Favole
Manico
Timore
Nipoti
Cenere
Fegato
Vetrina
Radice
Vagoni
Vapore
Miracoli
Facile
Felice
Vicini
Povera
Celebre
Nobili
Sicura
Simile
Comodo
Alpina
Rapidi
Navali
Ultime
Antica
Notevole
Ridicolo
Naturale
Cattivo
Rigido
Geloso
Divina
Diversa
Penosa
Severi
Gelida
Timido
Debole
Veloce
Valido
Solito
Comunale
Piccoli
Esteri
Feroce
Capace
Famosa
Morbido
Sincere
Tragico
Cretino
Erano
Vengono
Aveva
Vivere
Bollire
Sudava
Passano
Capire
Pareva
Furono
Caddero
Tentare
Toccare
Ridere
Salito
Bevono
Prestano
Morire
Volete
Trovare
Credere
Perdere
Tornare
Succede
Dicono
Cercano
Contano
Salvare
Supera
Devono
Pagare
Piantare
Guardano
Pensare
Mettere
Prendete
Recitare
Sapevano
Appariva
Accomodi.

*List 3*

Sedia/Bugia
Ovvio/Addio
Boria/Moria
Paggio/Leggio
Media/Mania
Madia/Malia
Sabbia/Pazzia
Begonia/Bigamia
Malaria/Funivia
Varia/Magia.
